# 24S-Hydroxycholesterol Correlates With Tau and Is Increased in Cerebrospinal Fluid in Parkinson's Disease and Corticobasal Syndrome

**DOI:** 10.3389/fneur.2018.00756

**Published:** 2018-09-07

**Authors:** Ingemar Björkhem, Kalicharan Patra, Adam L. Boxer, Per Svenningsson

**Affiliations:** ^1^Department of Laboratory Medicine Karolinska Institutet, Stockholm, Sweden; ^2^Department of Clinical Neuroscience Karolinska Institutet, Stockholm, Sweden; ^3^Memory and Aging Center, University of California, San Francisco San Francisco, CA, United States

**Keywords:** 24S-hydroxycholesterol, oxysterols, biomarkers, CSF, Parkinson's disease, corticobasal degeneration

## Abstract

24S-hydroxycholesterol (24OHC) and Tau are produced in neuronal cells and neurodegeneration leads to increased flux of both of them into cerebrospinal fluid (CSF). In the present study, CSF levels of 24OHC and 27S-hydroxycholesterol (27OHC) along with those of Tau, P-Thr^181^-Tau and Aβ_42_ were measured in patients with early Parkinson's disease (PD), Corticobasal syndrome (CBS), Corticobasal Degeneration (CBD), and controls. Using mouse models with increased or no formation of Tau protein and increased production of 24OHC, we have also tested the hypothesis that there is a direct association between neuronal turnover of 24OHC and Tau. The levels of 24OHC are increased, at a group level, in patients with PD or CBS. We found significant correlations between levels of 24OHC and Tau or P-Thr^181^-Tau in CSF from patients with PD, CBS or CBD. There were no similar correlations between 24OHC and Aβ_42_ in CSF from these patients. The neuronal levels of 24OHC were not altered in Tau knockout or Tau overexpressing mice. *Vice versa*, Tau species levels were not changed in Cyp46 overexpressing mice with increased neuronal levels of 24OHC. We conclude that the strongly correlative fluxes of 24OHC and Tau from neuronal cells to CSF are likely to be secondary to neurodegeneration and not due to direct interaction between the two factors. We suggest that this high correlation reflects a rapid neurodegeneration of specific neuronal subtypes with simultaneous release of 24OHC and Tau into the CSF.

## Introduction

In contrast to cholesterol itself its side-chain oxidized metabolites 24S-hydroxycholesterol (24OHC) and 27-hydroxycholesterol (27OHC) are able to pass the blood-brain barrier. 24OHC is exclusively formed in neurons and is continuously fluxed into the circulation (1). 27OHC is mainly formed in extracerebral tissues and organs, but there is a continuous flux of this oxysterol from the circulation into the brain (2). Neurodegeneration results in increased flux of 24OHC from neurons into CSF, possibly due to a direct release from the decomposing cells (1). Neurodegeneration also results in disruption of the blood-brain barrier and reduced capacity of the neuronal enzyme CYP7B1 to metabolize 27OHC resulting to increased 27OHC in CSF (2).

Evidence has accumulated that 24OHC in CSF can be used as a biomarker for neurodegeneration, particularly at early stages (3). Another commonly used biomarker for neurodegeneration is Tau. The role of this protein is to stabilize axonal microtubule by promoting tubulin assembly. Abnormal phosphorylation of neuronal Tau leads to destabilization and increased levels of Tau and phospho-Tau in CSF (4). In a previous study (5), we found a significant correlation between Tau or P-Thr^181^-Tau and 24OHC in CSF from patients with Alzheimer's disease and mild cognitive impairment. This finding was confirmed in a later study by another group (6).

Here, we have compared CSF levels of 24OHC and 27OHC along with those of Tau, P-Thr^181^-Tau and Aβ_42_ in patients with parkinsonism. Specifically, we have studied CSF samples from patients with Parkinson's disease (PD), which is a synucleinopathy, but associated with genetic polymorphisms regulation tau expression (7). We have also studied samples from patients with Corticobasal syndrome (CBS), a condition characterized by filamentous Tau inclusions in neurons and astrocytes (8, 9). Since CBS encompasses a diagnostically heterogeneous group of patients (8, 9), we also evaluated CSF from pathologically confirmed cases with Corticobasal Degeneration (CBD). Finally, we have tested the hypothesis that there is a direct association between neuronal turnover of 24OHC and Tau with use of mouse models with increased or no formation of Tau protein and increased production of 24OHC.

## Materials and methods

### Patients and CSF sampling

This study involved CSF samples from patients from the Neurology clinic, Karolinska University Hospital and the Memory Clinic, University of California, San Francisco (UCSF) Memory and Aging Center. All the investigations of the patients and the analyses of their CSF were approved by the ethic committees of the respective institutions. Informed consent was obtained from the subjects.

In experiment 1, CSF from controls (i.e., subjects with tension headache or benign parastesia), patients with early PD [diagnostic criteria, see (10)] or CBS [diagnostic criteria see (8)] were analyzed. In experiment 2, CSF from patients with pathologically confirmed CBD from UCSF was studied. The CBD diagnosis was made according to a previously described procedure (9). Demographic information about the different grups are presented in Table 1.

**Table 1:** Demographics of examined controls (Ctrl) and patients with Parkinson's Disease (PD), Corticobasal syndrome (CBS), and Corticobasal degeneration (CBD).

	**Age (years)**	**Gender (M:F)**	**Disease duration (years)**	**Levodopa equivalent dose (LED)**
Ctrl (*n* = 19)	58.2 ± 9.3 (45–82)	14:5	n/a	n/a
PD (*n* = 30)	63.9 ± 10.8 (38–89)	21:9	0	0
CBS (*n* = 11)	68.9 ± 5.4 (60–81)	7:4	2.5 ± 1.7 (0–6)	144 ± 240 (0–764)
CBD (*n* = 8)	65.8 ± 3.1 (62–70)	6:2	n/k	0

*Data are presented as the mean ± standard deviation and (range). n/a stands for not applicable and n/k for not known*.

The CSF samples were obtained by lumbar puncture and collected into polypropylene-tubes and subsequently centrifuged (1,300–1,800 × g, 4°C, 10 min). The supernatant was carefully pipetted off and dispensed in 500 μl aliquots before storage at −80°C. The time interval from collection to freezing was less than 60 min.

### Animal experiments

All the animal experiments were approved by the local Animal Experimentation Ethics Committee.

#### Mouse models with overexpressed or no tau levels

Twelve weeks old male wildtype, tau KO and hTau OE mice on a C57Bl6 background were used. Both mutant Tau lines are deficient of murine Tau, but hTau OE mice overexpress all six human Tau isoforms, leading to gradual Tau pathology and behavioral deficits (11). Mice were decapitated, cortical brain tissue samples dissected, immediately frozen at −80°C until analysis for oxysterol levels.

#### Mouse model with overexpressed CYP46 and high levels of 24OHC in circulation and brain

Ten weeks old male mice with overexpression of CYP46 under the β-actin promotor on a C57Bl6 background were used (12). CYP46 is an enzyme synthetizing 24OHC and the CYP46 OE mice have a 2-fold increase of 24OHC in the brain and a 4–6-fold increase in serum (12). The mice have no obvious behavioral phenotype. Cortical brain tissue was obtained as above and analyzed for Tau species.

### Analyses of oxysterols

The analyses of 24OHC and 27OHC in CSF and mouse brain tissue were performed by isotope dilution mass spectrometry as described previously (2, 3, 13). In one CSF sample from a CBS patient could only 24OHC, and not 27OHC, be measured.

### Analyses of tau species, Aβ_42_ and neurofilament

Tau, P-Thr^181^ Tau (commonly referred to as phospho-tau) and Aβ_42_ analyzes in CSF samples from Karolinska were made with enzyme-linked immunosorbent assay (ELISA) kits from Innogenetics NV Ghent Belgium. CSF samples from USCF were analyzed with the INNO-BIA AlzBio3 (Innogenetics, Ghent, Belgium) platform to measure Tau, P-Thr^181^ Tau and Aβ_42_ and the Uman Diagnostics ELISA kit (Umea, Sweden) to measure neurofilament (NFL).

Levels of total Tau, 4R Tau and P-Ser^202^ Tau in cortical brain tissue from wildtype and CYP46 OE mice were determined by Western blotting and chemiluminiscence as previously described (14). The primary antisera were kind gifts from Drs Peter Davies and Rohan da Silva.

### Statistics

Data are presented as mean ± S.D. Data was tested for normality using the Kolmogorov–Smirnov test. When two groups were compared, unpaired Student's *t*-test was used. When more than two groups were compared, statistical analyses were made with one-way ANOVA followed by Dunnet's test or Kruskal Wallis test followed by Dunn's test. Correlation analyses were made with Pearson's test followed by *t*-tests. All statistical analyses were made with GraphPAD Prism (GraphPAD Prism 5.0).

## Results

### Levels of 24OHC and 27OHC in patients with PD or CBS

**Figure 1:**
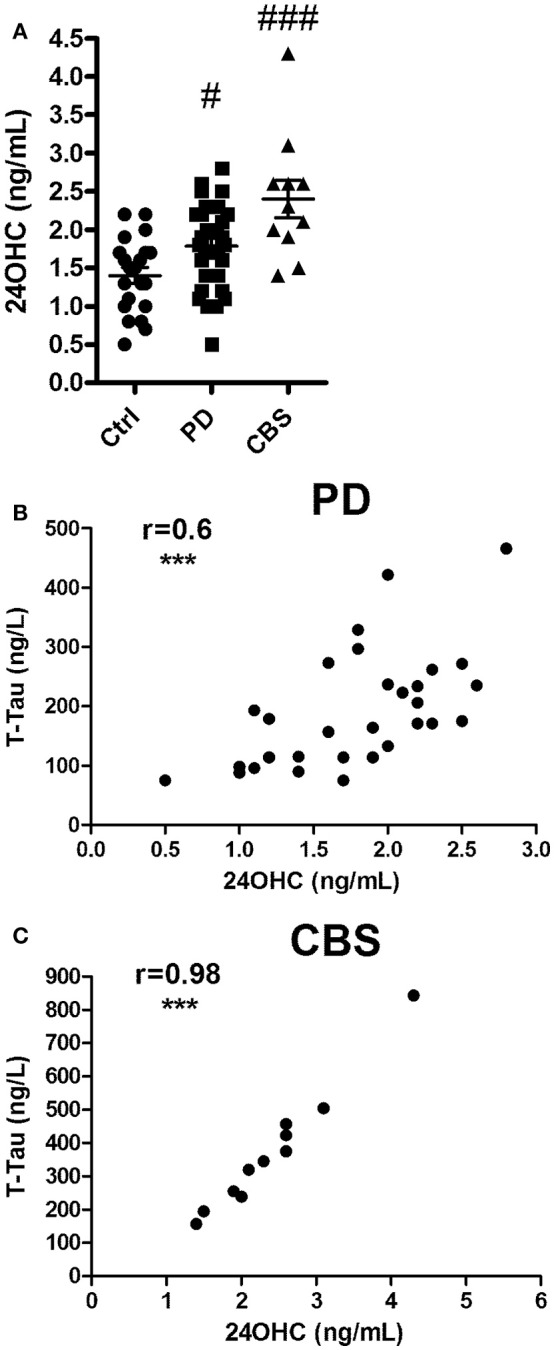
**(A)** Levels of 24OHC in CSF from controls or patients with Parkinson's disease (PD) or Corticobasal syndrome (CBS). **(B,C)** Correlations between CSF levels of 24OHC and total Tau in PD **(B)** and CBS **(C)** patients. In **(A)**, ^#^*p* < 0.05, ^###^*p* < 0.001 vs. control. In **B,C**, *r* values indicate Pearson correlations and ^***^p < 0.001 significance.

Patients with PD or CBS had significantly [*F*_(2, 61)_ 10.6; *p* < 0.001] higher levels of 24OHC in CSF than those of the control subjects (*p* < 0.05 and *p* < 0.001, respectively) (Figure 1A). The data for 27OHC in CSF was not normally distributed, but showed significant (Kruskal Wallis value 10.5) difference. Pairwise comparisons showed that the levels were significantly higher in the patients with CBS compared to controls (*p* < 0.01) (Supplementary Figure 1A).

#### Correlations between levels of 24OHC or 27OHC and tau, P-Thr^181^ tau and Aβ42 in CSF from patients with PD or CBS

Significant correlation was observed between levels of 24OHC and Tau (*r* = 0.6, *p* < 0.001) in CSF from patients with PD (Figure 1B). A similar correlation was observed between 24OHC and P-Thr^181^ Tau (*r* = 0.62, *p* < 0.001) (Supplementary Figure 2A), but not between 24OHC and Aβ42 (*r* = 0.21, *p* = 0.27) (Supplementary Figure 2C). There was a lower, but significant (*r* = 0.38, *p* = 0.04 vs. *r* = 0.39, *p* = 0.03), correlation between 27OHC and Tau and P-Thr^181^ Tau in CSF from PD patients (Supplementary Figures 1B, 2B). There was no significant (*r* = 0.27, *p* = 0.14) correlation between 27OHC and Aβ42 in these patients (Supplementary Figure 2D).

There was a very high correlation between 24OHC and Tau (*r* = 0.98, *p* < 0.0001) as well as P-Thr^181^ Tau (*r* = 0.89, *p* < 0.001) in patients with CBS (Figure 1C, Supplementary Figure 3A). There was no significant (*r* = −0.16, *p* = 0.64) correlation between 24OHC and Aβ42 (Supplementary Figure 3C). There were no significant correlations between 27OHC and Tau (*r* = 0.32, *p* = 0.37), P-Thr^181^ Tau (*r* = −0.37, *p* = 0.3) or Aβ42 (*r* = 0.46, *p* = 0.18) in the CBS patients (Supplementary Figures 1C, 3B,D).

**Figure 2:**
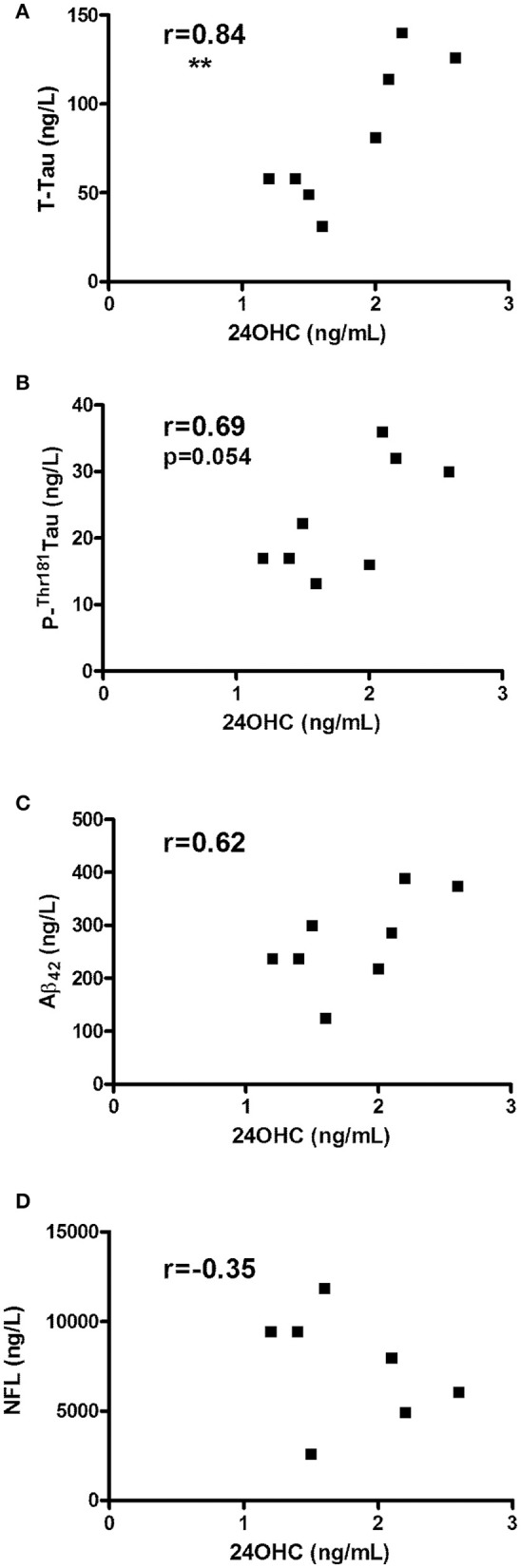
Correlations between CSF levels of 24OHC and total Tau **(A)**, P-Thr^181^-Tau **(B)**, Aβ_42_ **(C)** or NFL **(D)** in patients with corticobasal degeneration (CBD). *r* values indicate Pearson correlation and ^**^p < 0.01 significance.

Since CBS encompasses a diagnostically heterogeneous group of patients (8, 9), we also evaluated CSF from pathologically confirmed cases with CBD. In accordance with obtained data from CBS patients, CBD patients showed a significant correlation between 24OHC and Tau (*r* = 0.84, *p* = 0.008) (Figure 2A) and a strong trend with P-Thr^181^ Tau (*r* = 0.69, *p* = 0.054) (Figure 2B). There was no significant (*r* = 0.62, *p* = 0.10) correlation between 24OHC and Aβ42 (Figure 2C). Measures of NFL was also available from these CBD patients, but they did not show (*r* = −0.35, *p* = 0.44) a significant correlation to 24OHC (Figure 2D). There were no significant correlations between 27OHC and Tau (*r* = 0.29, *p* = 0.48), P-Thr^181^ Tau (*r* = −0.14, *p* = 0.75), Aβ_42_ (*r* = 0.17, *p* = 0.69) or NFL (*r* = −0.24, *p* = 0.61) (Supplementary Figures 4A–D).

There was no correlation between 24OHC and Tau (*r* = 0.29, *p* = 0.38), P-Thr^181^ Tau (*r* = 0.05, *p* = 0.89) or Aβ42 (*r* = 0.06, *p* = 0.85) in control subjects. There was no significant correlation between 24OHC levels and age in controls (*r* = 0.18, *p* = 0.45), PD (*r* = 0.01, *p* = 0.95) or CBD (*r* = 0.09, *p* = 0.83) subjects, whereas there was a positive correlation in CBS patients (*r* = 0.81, *p* = 0.003).

### Mouse experiments designed to test the hypothesis that there is a direct interaction between 24OHC and tau turnover in the brain

Based on the strong correlation between 24OHC levels and Tau in CSF, we used mutant mouse models to examine whether there could be a direct relation in their neuronal production. The hypothesis that increased neuronal production of 24OHC affects levels of Tau protein was tested in mice overexpressing CYP46. It is known that these mice have a two-fold increased levels of 24OHC in the brain (10). However, there were no significant differences between wildtype and CYP46OE mice in their levels of total Tau, P-Ser^202^ Tau (CP-13) or 4R-Tau in cortical brain tissue (Supplementary Figure 5). *Vice versa*, the hypothesis that primary changes in the levels of Tau are able to affect levels of 24OHC in the brain was tested with use of mice with no (Tau KO mice) or increased (Tau OE) levels of Tau. As shown in Supplementary Figure 6A, the levels of 24OHC were not significantly [*F*_(2, 15)_ 0.07] different between these groups. There was neither any significant (Kruskal Wallis value 3.8) alterations in the levels of 27OHC in the genetically modified Tau mice (Supplementary Figure 6B).

## Discussion

There is a continuous production of 24OHC in neuronal cells and a flux of this oxysterol from the brain into the circulation (1). A neurodegeneration will reduce this production resulting in slightly reduced levels of 24OHC in the circulation (3). The changes are however small and are difficult to use diagnostically. In contrast a neurodegeneration results in increased levels of 24OHC in CSF most probably due to a release from dying neuronal cells. This increase is sufficiently high to be used diagnostically (15). Assuming that a considerable part of the 24OHC and Tau in CSF is released from dying neuronal cells, a correlation between these two parameters can be expected in neurodegenerative disorders. Evidently, there was a strong correlation between 24OHC and Tau in CSF from PD patients. The CSF was collected at an early stage of PD and the patients had no medication against PD. There was also a significant but weak correlation between Tau and 27OHC in the PD patients. The flux of 27OHC is likely to be dependent upon the rate of metabolism of this oxysterol by the enzyme CYP7B1 (2). The latter enzyme is mainly present in neuronal cells and a reduction of the number of these cells can be expected to increase the level of 27OHC into CSF. It should be pointed out that in contrast to 27OHC, 24OHC is not a substrate for CYP7B1. In view of this a considerably lower correlation between 27OHC and Tau can be expected. It should also be pointed out that due to the extensive metabolism, the levels of 27OHC in the brain are much lower than the corresponding levels of 24OHC (16).

The strongest finding here is the high correlation between 24OHC and Tau or P-Thr^181^-Tau in CSF from patients with CBS and CBD. CBD is caused by accumulation predominantly of 4R Tau. The underlying CBD pathology gives rise to a variety of clinical presentations that encompass CBS, characterized by levodopa resistant asymmetric dystonia or rigidity, bradykinesia and myoclonus along with cortical symptoms such as apraxias, speech difficulties and alien limb phenomenona. However, CBD also includes a syndrome clinically similar to progressive supranuclear palsy (PSPS-CBD), a frontotemporal behavioral variant (FTD-CBD) and a variant with progressive non-fluent aphasia (PNFA-CBD). Due to this heterogeneity in presentation, a clinical diagnosis of CBD is often difficult and many patients will receive an *ante mortem* diagnosis that is altered upon *post mortem* examination (8, 9). In our first experiment we examined CSF from living patients with a typical CBS presentation, but could later verify the strong correlation between 24OHC and Tau in another cohort of pathologically confirmed CBD patients. Another difference between these cohorts were that the patients in the CBD cohort were not on any medication against parkinsonism which could potentially influence oxysterol levels.

The very high correlation between 24OHC and Tau led us to test the hypothesis that there is a direct interaction between the neuronal production of the two factors. Indeed, based on experiments with mouse models the possibility has been discussed that there may be a causal link between CYP46A1 protein content and memory impairment that result from Tau pathology (17).

However, experiments with a mouse model with high levels of 24OHC and mouse models with increased or no levels of Tau did not give support for this hypothesis. Thus, 24OHC is not likely to be a driving force for increased production of Tau and, *vice versa*, Tau is not likely to directly determine the production of 24OHC. It is noteworthy that young mice were used in our studies to avoid indirect influence of aging, but it may also turn out that reciprocal changes in 24OHC and Tau are only evident in older mice. Nonetheless, it seems likely that the correlation between the two factors in the patients is secondary to neurodegeneration, in PD as well as CBD patients. In theory, a high correlation between 24OHC and Tau can be expected if there is a very rapid decomposition of individual neuronal cells with a simultaneous release of both 24OHC and Tau. Accordingly, a higher correlation was seen in CBS/CBD than in PD patients, likely to reflect the more aggressive neurodegeneration in CBD/CBS.

In conclusion, CSF levels of 24OHC are elevated, at group level, in PD, CBS, and CBD, and show a very strong correlation to Tau. Future studies will evaluate whether 24OHC and Tau interact synergistically in pathophysiological events underlying PD or CBD. It will also be interesting to study whether there is a correlation between 24OHC and α-synuclein and to measure 24OHC in the same individual at different disease stages in longitudinal cohort studies.

## Ethics statement

This study was approved by the Research Ethics Committee of the Karolinska University Hospital and University of California, San Francisco. All subjects gave a written informed consent in accordance with the Declaration of Helsinki before the measurements.

## Author contributions

IB: study planning, sample measurements, and manuscript writing. KP: sample measurements, data analysis, and manuscript writing. AB: patient and sample recruitment and manuscript writing. PS: study planning, data analysis, and manuscript writing.

### Conflict of interest statement

The authors declare that the research was conducted in the absence of any commercial or financial relationships that could be construed as a potential conflict of interest.

## Supplementary material

Supplementary Figure 1 **(A)** Levels of 27OHC in CSF from controls or patients with Parkinson's disease (PD) or Corticobasal syndrome (CBS). **(B,C)** Correlations between CSF levels of 27OHC and total Tau in PD **(B)** and CBS **(C)** patients. In A, ^#^*p* < 0.05 vs. control. In **B,C**, *r* values indicate Pearson correlations and ^*^*p* < 0.05 significance.

Supplementary Figure 2 Correlations between CSF levels of 24OHC **(A,C)** or 27OHC **(B,D)** and P-Thr^181^-Tau **(A,B)** or Aβ_42_ **(C,D)** in patients with PD. *r* values indicate Pearson correlation and ^*^*p* < 0.05, ^***^*p* < 0.001 significance.

Supplementary Figure 3 Correlations between CSF levels of 24OHC **(A,C)** or 27OHC **(B,D)** and P-Thr^181^-Tau **(A,B)** or Aβ_42_ **(C,D)** in patients with CBS. *r* values indicate Pearson correlation and ^***^*p* < 0.001 significance.

Supplementary Figure 4 Correlations between CSF levels of 27OHC and total Tau **(A)**, P-Thr^181^-Tau **(B)**, Aβ_42_ **(C)** or NFL **(D)** in patients with CBD. *r* values indicate Pearson correlations.

Supplementary Figure 5 Relative levels of total Tau, P-Ser^202^-Tau (“CP13”) and 4-repeat Tau in cortical brain tissue of wildtype (WT) mice and mice with an overexpression of CYP46 and increased levels of 24OHC.

Supplementary Figure 6 Levels of 24OHC **(A)** and 27OHC **(B)** in cortical brain tissue of wildtype (WT) mice or with increased (Tau OE) or no (Tau KO) levels of Tau.
