# Supplementary figures and images for: 24S-Hydroxycholesterol Correlates With Tau and Is Increased in Cerebrospinal Fluid in Parkinson's Disease and Corticobasal Syndrome
Source: Front Neurol. 2018 Sep 7;9:756. doi: 10.3389/fneur.2018.00756 (PMC6137204; doi:10.3389/fneur.2018.00756)

**A**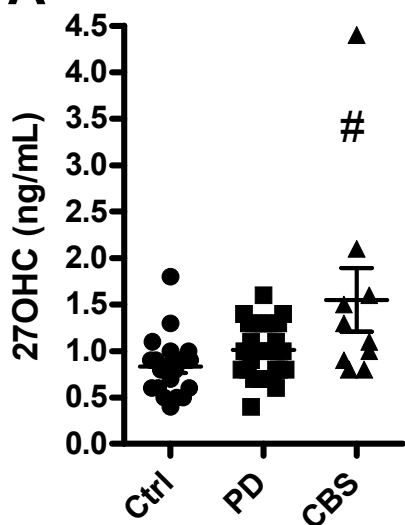**B**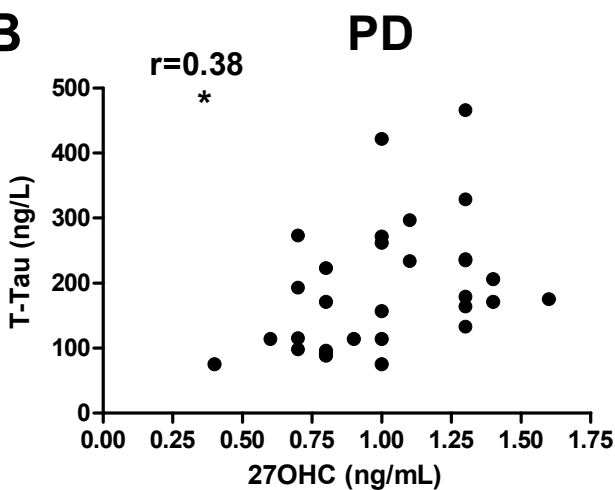**C**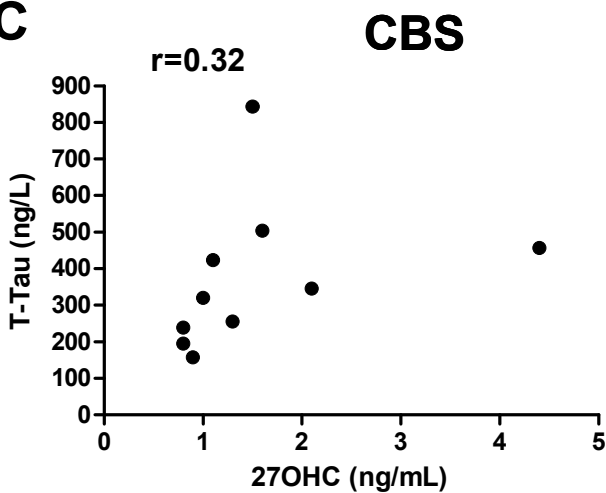

Supplement: Supplementary Figure 1 — (A) Levels of 27OHC in CSF from controls or patients with Parkinson's disease (PD) or Corticobasal syndrome (CBS). (B,C) Correlations between CSF levels of 27OHC and total Tau in PD (B) and CBS (C) patients. In A, #p < 0.05 vs. control. In B,C, r values indicate Pearson correlations and *p < 0.05 significance. [file Data_Sheet_1.PDF]

**A**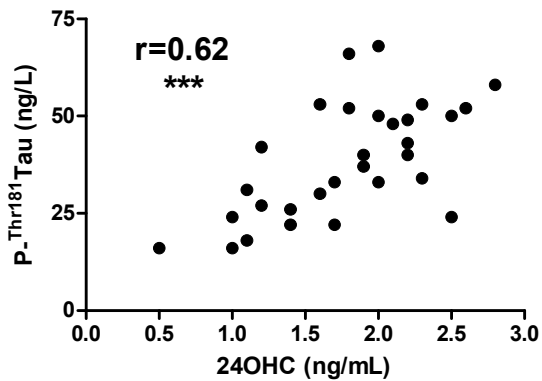**B**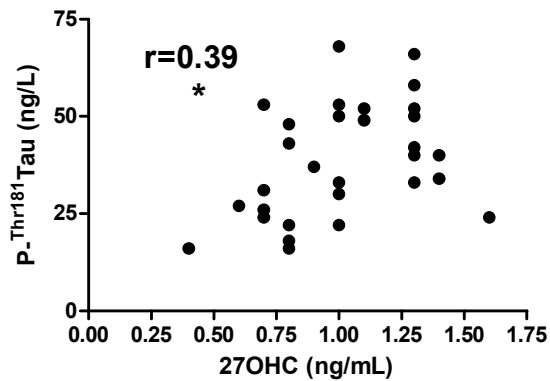**C**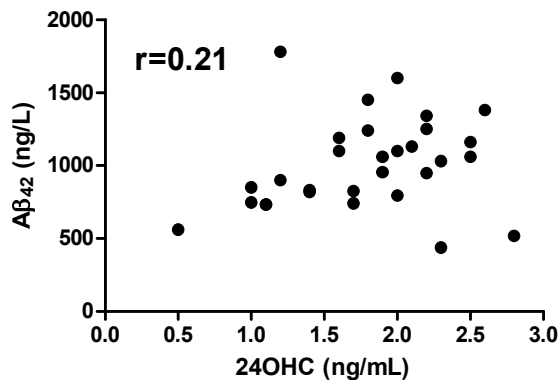**D**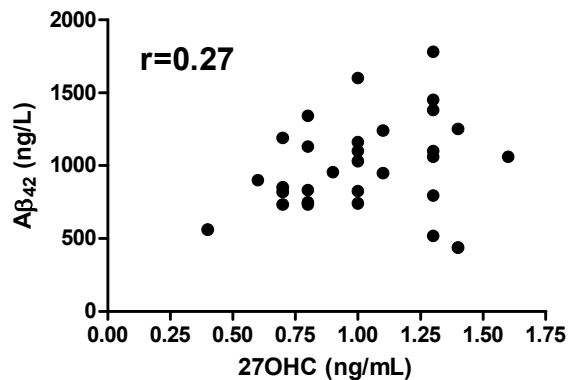

Supplement: Supplementary Figure 2 — Correlations between CSF levels of 24OHC (A,C) or 27OHC (B,D) and P-Thr181-Tau (A,B) or Aβ42 (C,D) in patients with PD. r values indicate Pearson correlation and *p < 0.05, ***p < 0.001 significance. [file Data_Sheet_2.PDF]

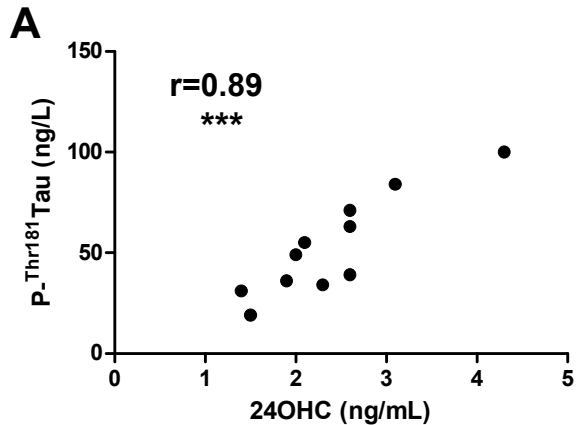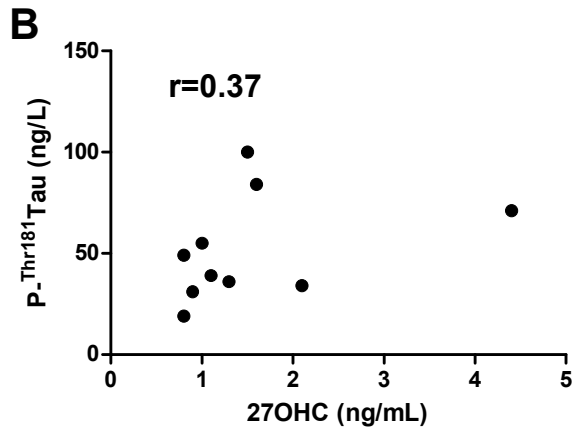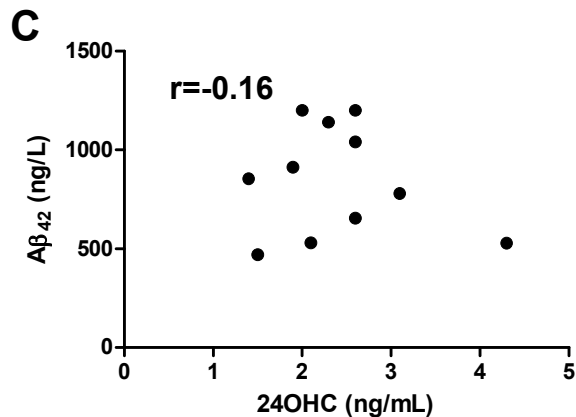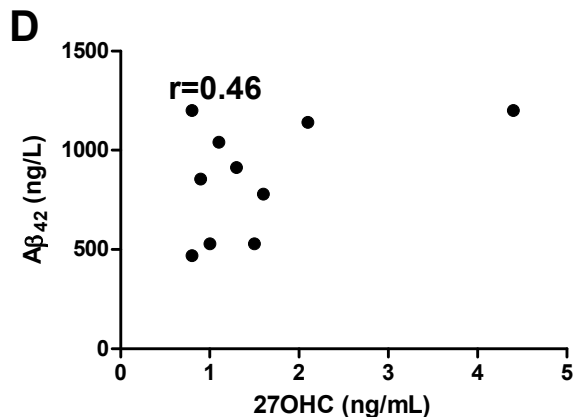

Supplement: Supplementary Figure 3 — Correlations between CSF levels of 24OHC (A,C) or 27OHC (B,D) and P-Thr181-Tau (A,B) or Aβ42 (C,D) in patients with CBS. r values indicate Pearson correlation and ***p < 0.001 significance. [file Data_Sheet_3.PDF]

**A**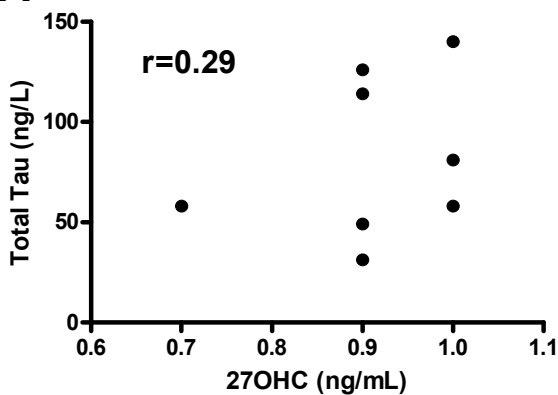**B**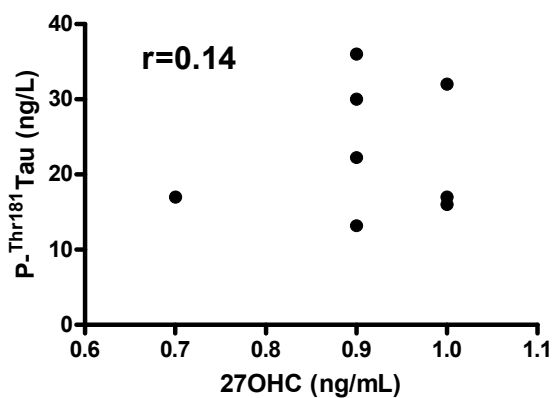**C**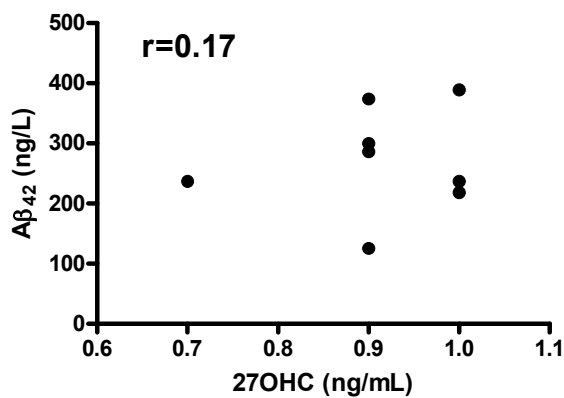**D**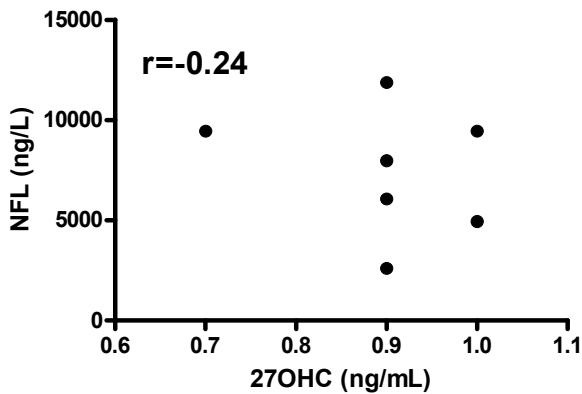

Supplement: Supplementary Figure 4 — Correlations between CSF levels of 27OHC and total Tau (A), P-Thr181-Tau (B), Aβ42 (C) or NFL (D) in patients with CBD. r values indicate Pearson correlations. [file Data_Sheet_4.PDF]

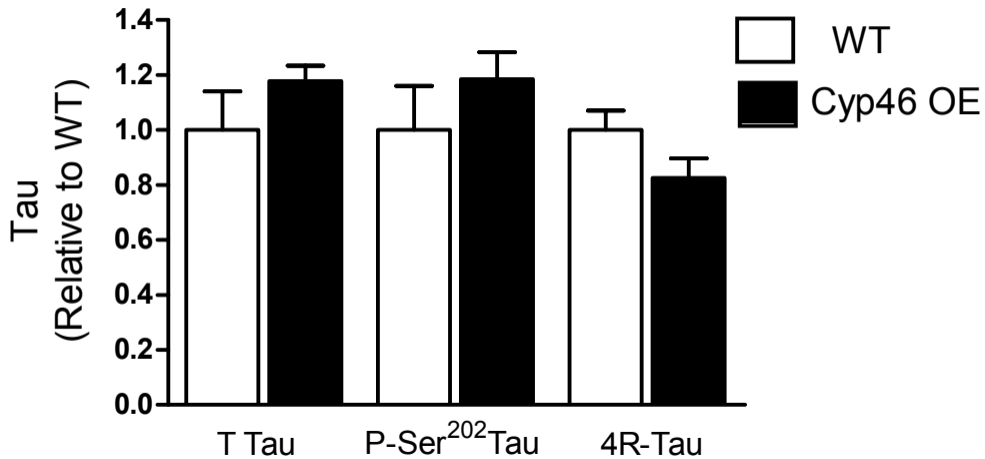

Supplement: Supplementary Figure 5 — Relative levels of total Tau, P-Ser202-Tau (“CP13”) and 4-repeat Tau in cortical brain tissue of wildtype (WT) mice and mice with an overexpression of CYP46 and increased levels of 24OHC. [file Data_Sheet_5.PDF]

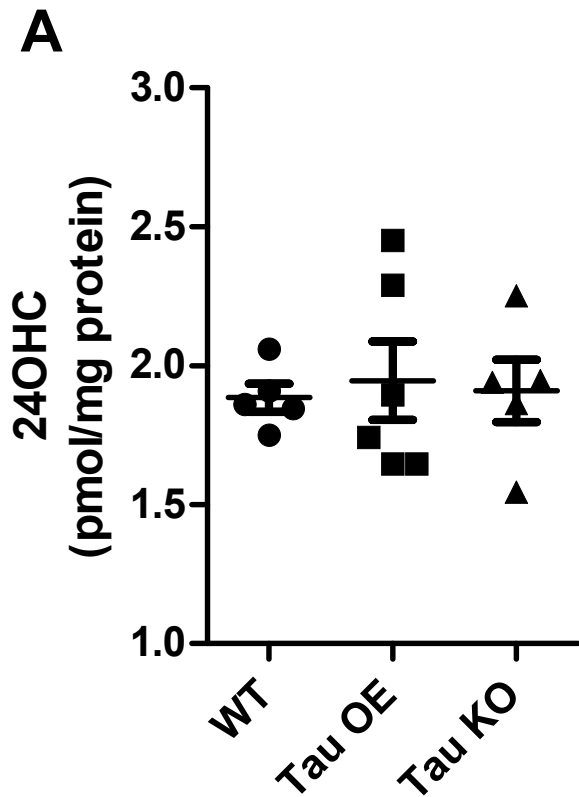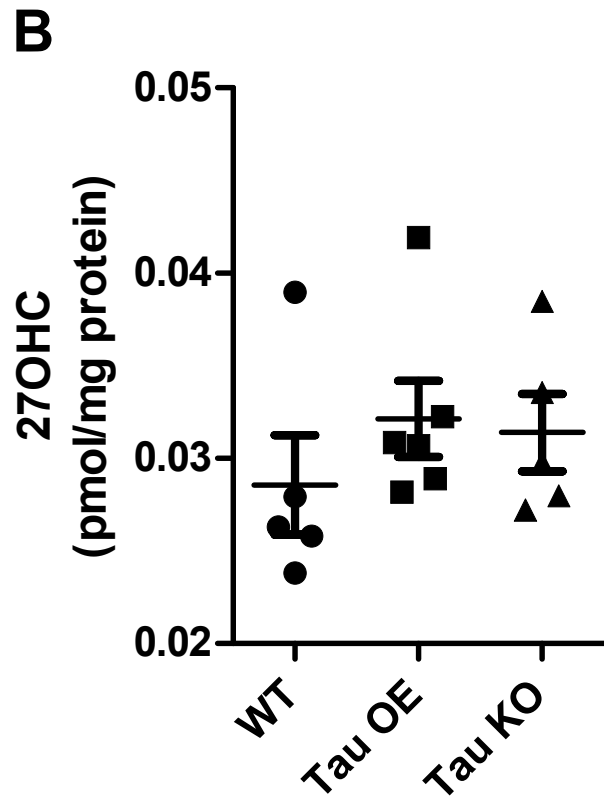

Supplement: Supplementary Figure 6 — Levels of 24OHC (A) and 27OHC (B) in cortical brain tissue of wildtype (WT) mice or with increased (Tau OE) or no (Tau KO) levels of Tau. [file Data_Sheet_6.PDF]
